# Valuing pharmacists as members of the eye team

**Published:** 2023-05-22

**Authors:** Parvesh Patel

**Affiliations:** Community Pharmacist: London, UK.

**Pharmacists have extensive knowledge about the ever-increasing number of eye medicines available; their input is vital to protect patients and ensure effective treatment.**

**Figure:**
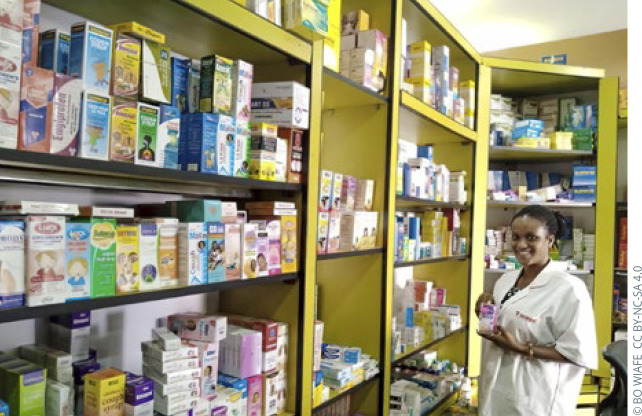
Every contact between pharmacist and patient is an opportunity to improve patients? eye health. ghana

Pharmacists are valuable members of the eye team. They can support the eye care team to make effective, patient-centred decisions by sharing their extensive knowledge about:

- the range of medicines available locally for eye conditions
- the interactions between different medicines
- how to improve adherence, e.g., by suggesting a change in the type of bottle being used, or a change in formulation (e.g., with a preservative-free product to reduce stinging sensation).

The role of the hospital pharmacist as part of a multidisciplinary team is usually understood and accepted. However, the role of the community pharmacist may not be well understood.

In the community setting, the pharmacy is often seen as a simply the location at which patients collect their medication. The community pharmacy is, however, an essential point of contact for primary health care, including primary eye care. Patients come to seek advice about a wide range of conditions and about over-the-counter or prescribed medication.

Community pharmacists play an essential clinical role in assessing the nature of patients' primary concerns (e.g., is it allergic, infective, or acute?) and deciding whether they need to be referred. Pharmacists can also offer support, information, and reassurance to their patients.

## Good working relationships

Effective teamwork between community pharmacists and other members of the eye team is essential to ensure patient safety and improve patient outcomes, so it is therefore important to create opportunities for collaboration and to set up effective communication channels between community pharmacists and the eye team. Here are a few ideas.

### Prescription checking

- Recognise that prescription errors are often picked up by pharmacists, who are specifically trained to do so. Acknowledge that this is in the best interests of both patients and doctors. Acknowledge that this is in the best interests of patients and doctors. Respect pharmacists’ role and training.
- Share the eye team's contact details with community pharmacists, so that it is easy and convenient for pharmacists to resolve queries and potential errors.
- When giving patients a prescription to take to the pharmacy, it may be helpful to attach a copy of their hospital or discharge notes (containing their diagnosis, the medication they have been prescribed, and the dates of any follow-up appointments). This will make it easier for the pharmacist to detect inconsistencies or prescription errors. (In the UK, patients who leave the hospital receive a ‘discharge summary’ which is shared with pharmacists via an electronic medical records system; this allows pharmacists to follow up on the prescribing and dispensing of medication for each patient.)

### Referral and feedback

Set up a referral and feedback mechanism between the eye clinic and community pharmacists.

- Ensure pharmacists have up-to-date information about clinic days and times, so patients don't have the expense of a wasted journey.
- Give feedback to pharmacists who refer patients to you. For example, you can thank them for referring the patient, confirm whether or not they were right to refer them, and offer support or guidance to improve future referrals.
- You could be proactive and give all the community pharmacists in the area a set of referral forms with your hospital or clinic details and space for them to add their contact details, the reason for referral, and to indicate whether or not this is an emergency.
- The clinic administrators can also send the patient's discharge information directly to the pharmacist.

### Training

The multidisciplinary eye team does not stop at the hospital door – it extends out to the community. Some ideas for bringing community pharmacists into closer contact with the hospital-based eye team include the following.

- Offer training sessions for community pharmacists and outreach nurses/eye care workers. Offer sessions on basic eye care, specialist sessions, e.g., eye infections or glaucoma care, or practical sessions such as referral guidelines and procedures
- Invite pharmacists to share their knowledge about the latest eye medications or formulations. This is a great opportunity for learning for all members of the multidisciplinary eye team to learn about the role of community pharmacists/nurses.

### Care for long term eye conditions

- For chronic eye conditions such as glaucoma, the community pharmacist may see the patient more often than the ophthalmologist. Sharing the patient's care plan with the pharmacist can help, as every contact they have with the patient is an opportunity for them to support adherence and safety, and to reinforce key messages.
  Pharmacy in eye care is an ever-growing industry, with new drugs and formulations coming onto the market every few months. Pharmacists undergo many years of training, and their input is vital to ensure patient safety and effective treatment. They can advise clinicians about potential interactions, suitability, availability, and alternatives. Therefore, collaborating closely with community pharmacists will help eye care providers to offer effective and safe treatment options in both hospital and community settings.
